# Transporting the effect of the ASSIST school‐based smoking prevention intervention to the Smoking, Drinking and Drug Use Among Young People in England Survey (2004–2021): A secondary analysis of a randomized controlled trial

**DOI:** 10.1111/add.70141

**Published:** 2025-07-07

**Authors:** James White

**Affiliations:** ^1^ Centre for Trials Research Cardiff University Cardiff UK

**Keywords:** generalizability, prevention, randomized controlled trial, real world evidence, smoking, transportability

## Abstract

**Aims:**

To conduct exploratory analyses into the transported effect of the ASSIST (A Stop Smoking in Schools Trial) school‐based smoking prevention intervention on weekly smoking in young people between 2004 and 2021.

**Design:**

Secondary analysis of a cluster randomized control trial (cRCT).

**Setting:**

England and Wales.

**Participants:**

ASSIST trial participants comprised 8756 students aged 12–13 years in 59 schools assigned using stratified block randomization to the control (29 schools, 4193 students) or intervention (30 schools, 4563 students) condition. The target population was represented by 12–13‐year‐old participants in the Smoking, Drinking and Drug Use Among Young People in England Survey (SDDU) in 2004 (*n* = 3958), 2006 (*n* = 3377), 2014 (*n* = 3145), 2016 (*n* = 4874) and 2021 (*n* = 3587), which are randomly sampled school‐based surveys with student response rates varying between 85% and 93%.

**Intervention and comparator:**

The ASSIST intervention involved 2 days of off‐site training of influential students to encourage their peers not to smoke over a 10‐week period. The control group continued with their usual education.

**Measurements:**

The outcome was the proportion of students who self‐reported weekly smoking 2 years post‐intervention.

**Findings:**

The prevalence of weekly smoking at the 2‐year follow‐up in the ASSIST trial in 2004 was 4.1%, 49.5% of students were girls, and 7.8% ethnic minorities. In the SDDU in 2004, the prevalence of weekly smoking was 3.6%, 47.6% students were girls and 14.4% ethnic minorities and in 2021 0.2% were weekly smokers, 48.6% girls and 27.8% ethnic minorities. The odds ratio of weekly smoking in the ASSIST trial in 2004 was 0.85 [95% confidence interval (95% CI) = 0.71–1.02]. The estimated odds ratio in the SDDU target population in 2004 was 0.90 (95% CI = 0.72–1.13), in 2014 was 0.89 (95% CI = 0.70–1.14), and by 2021 was 0.88 (95% CI = 0.60–1.28). The confidence interval ratio was used to estimate precision in the transported estimates in the target population and was 1.57 in 2004, 1.63 in 2014 and 2.13 in 2021, reflecting increasing uncertainty in the effect of ASSIST over time. Subgroup analyses showed effects were comparable when restricted to only English schools in the ASSIST trial.

**Conclusions:**

These exploratory analyses indicate the effect of the ASSIST school‐based smoking prevention intervention reported in the original trial may not have been replicated in the target population over the 17‐year period of its licensing and roll out.

## INTRODUCTION

Randomized controlled trials (RCTs) are the gold standard in evaluating the effect of interventions. The commissioners of health services often rely on the results from RCTs to inform their spending decisions. However, the results from trials may not generalize when participants do not adequately reflect the intended target population [1, 2, 3]. In recent years, a distinction has been made between different types of problems with external validity [4]. *Generalizability* is the extent to which study results apply to the population from which the study sample was drawn. *Transportability* is whether study results apply to a different target population. Transportability may be a particular issue if the trial was conducted in a different country, time or population, and where the effects are ‘contextually dependent’ [5]. Variations in usual practice or care, drivers of implementation (e.g. existing burden on intervention delivery staff) and prognostic characteristics in participants may change the extent to which effects are replicated in different populations, or in the same population over time [6, 7].

Transportability provides an efficient approach for extending the results of a study to an external target population [8]. The benefit being that users of evidence can estimate what the results would be if the study was conducted in their target population without having to repeat the study. At its simplest, the assumptions underpinning transportability require that all variables that modify the effect of the intervention and differ in distribution in the study and target population are measured and accounted for [8, 9]. To date, transportability methods have been applied to transport treatment effects to target populations [10, 11] to understand heterogeneity between trial sites [12] or population subgroups within a trial [13], but not the same target population over time.

Here, we use an example from A Stop Smoking in Schools Trial (ASSIST), which evaluated a school‐based smoking prevention intervention, to illustrate how to apply transportability theory to transport the effects estimated in a study to an external target population over time. The ASSIST RCT ended in 2002, with the intervention found to be effective in reducing the prevalence of weekly smoking in students aged 12–13 years [14]. The intervention was recommended in the UK National Institute for Health and Care Excellence (NICE) guidance [15], was licensed through a not‐for‐profit organization [16] and has been delivered to over 160 000 students, with a version available in French and an adaptation underway in Bogota [16]. Our exploratory analyses estimate the extent to which the effect of ASSIST would have been found in a target population of English adolescents over a 17‐year period since the trial ended.

## METHOD

### Design

The ASSIST study was a two‐arm cluster randomized controlled trial (cRCT) conducted in schools in the West of England and South East Wales. Full details of the design of ASSIST and data collection methods can be found elsewhere (ISRCTN 55572965) [17]. In 2001, 223 secondary schools were invited to participate. One hundred and twenty‐seven schools expressed an interest and were visited, and 113 agreed to participate. Sixty‐six schools were randomly sampled from these 113 and stratified using publicly available data or the information provided by schools, by: country (England or Wales); type of school (independent or state); mixed or single sex; English or Welsh speaking; year‐group size above the median for schools in the sampling frame (<200 or ≥200 students); and whether the level of entitlement to free school meals in each school, a measure of household‐level socio‐economic disadvantage, was above or below the national median school‐level entitlement of 19%. Of these 66 schools, 59 signed an agreement to be randomized. The current analysis was not proposed in the study protocol and uses data gathered at baseline (September 2001–February 2002) and at the final 2‐year follow‐up (November 2003–May 2004).

To estimate a target population we used the 2004, 2006, 2014, 2016 and 2021 Smoking, Drinking and Drug Use Among Young People in England Survey (SDDU). Full details of the study design, sampling and data collection methods can be found elsewhere [18, 19, 20, 21, 22]. The SDDU is a biennial cross‐sectional survey of UK students in years 7–11 (mostly 11–15 years of age) in a random sample of schools in England. The focus of the study cycles through alcohol, tobacco and illicit drugs between surveys, and thus some SDDU surveys ask fewer questions on smoking behaviour. The SDDU school‐level response rate for the five surveys used were, in chronological order, 70%, 65%, 40%, 28% and 12%. The corresponding student‐level response rates were 89%, 85%, 87%, 93% and 92%. Data from the SDDU was accessed through the UK data archive [23]. This article adheres to the Consolidated Standards of Reporting Trials (CONSORT) guidelines on the reporting of cRCTs [24].

### Procedures

In the ASSIST trial, stratified block randomization was used, with the strata defined by the same criteria as the random sampling. Written consent was obtained from parents on an opt‐out basis and students provided written assent. The Multi‐Centre Research Ethics Committee for Wales reviewed the ASSIST trial protocol and judged it as meeting ethically acceptable standards. In the SDDU, written consent was obtained from parents on an opt‐out basis and students provided verbal or implied assent (i.e. students were told participation was voluntary and by completing the survey they provided consent). Ethics committees at the National Centre for Social Research reviewed and approved the methods of the SDDU surveys conducted in 2004, 2006 and 2014, and Ipsos approved the methods of the SDDU surveys conducted in 2016 and 2021.

### Intervention

The ASSIST intervention was an informal school‐based peer‐led smoking prevention intervention delivered via UK year‐8 students’ (aged 12–13 years) social networks. In ASSIST, influential students are identified and trained to diffuse non‐smoking information and norms, principally through conversations with their school friends. Table S1 describes the five stages of the intervention.

### Measures and outcomes

At baseline, students in the ASSIST trial were asked to complete a questionnaire that included questions on their age, ethnicity (white, mixed race, Asian or Asian British, black or black British, Chinese, other), gender identity (boy, girl), the family affluence scale (0–6) [25] and smoking behaviour. Students at 12 intervention and 12 control schools provided a saliva sample for cotinine analysis at the 2‐year follow‐up to minimize reporting bias [26].

Students in the SDDU surveys self‐reported their gender identity (boy, girl), smoking status (never smoked, occasional, experimental, ex‐smokers, weekly smokers who smoked one or more cigarettes a week), ethnicity (white, minority), age (12 or 13 years of age), age when they first smoked a cigarette (never, 0–13 years of age), whether they lived with a smoker (yes, no) and whether they had school lessons about smoking cigarettes in the past 12 months (had a lesson; not had a lesson; I do not know).

The ASSIST trial and the SDDU surveys were administered in schools and students self‐reported on the same questions about whether they had ever smoked, how often they smoked and the number of cigarettes they smoked per week. The primary outcome was weekly smoking defined as whether participants had smoked at least one cigarette a week or not. For simplicity, we harmonized the trial and target population data so that 11 survey participants who were 11 years old were re‐coded as being 12 years old, and eight survey participants who were 14 years old were re‐coded as being 13 years old, to match the ages of the ASSIST trial participants. The 2016 and 2021 SDDU surveys did not focus on smoking, so around half the students were directed to questions that did not include the question on whether they resided with a smoker. The responses to this question for participants who were unable to answer it were coded as missing.

### Statistical methods

Our goal was to estimate the intention to treat (ITT) odds ratio (OR) for weekly smoking at the 2‐year follow‐up between those randomized to ASSIST or to usual practice in 2004, 2006, 2014, 2016 and 2021. To identify the target parameters within the ASSIST trial population, we assume conditional treatment exchangeability and treatment positivity (see Box 1). Conditional treatment exchangeability assumes that there is no confounding of the association between treatment assignment and weekly smoking incidence in the ASSIST study population. Treatment positivity asserts that there should be a non‐zero probability that any participant receives the treatment. Both assumptions are met by randomization within the ASSIST trial [27].

BOX 1 **Assumptions underpinning transportability.**

• Conditional treatment exchangeability: there is no unmeasured confounding of the treatment–outcome relationship. The causal effect in the study population (i.e. the ASSIST trial in the current example) must be valid.

• Positivity of treatment assignment: each participant in the study has a positive probability of receiving the treatment. This requires that for every individual in the target population, there exist comparable individuals in the study population in the treatment and control conditions.

• Conditional population exchangeability and population positivity: after adjusting for relevant covariates, the treatment–outcome relationship in the study population can be assumed to hold in the target population. This requires that all variables that modify the effect of the intervention and differ in distribution in the study and target population are measured and accounted for.

**FIGURE 1:**
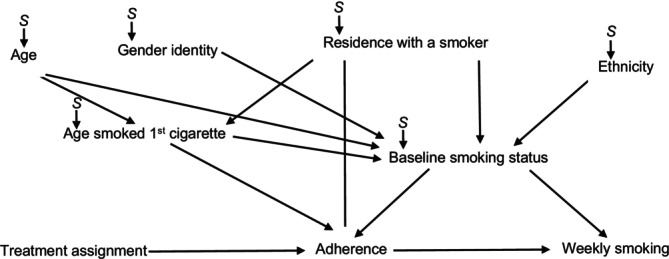
Proposed selection diagram representing the assumed causal model within the ASSIST trial (2001–2004) and the assumed differences between the study population and each target population (2004–2021). *S* = selection node.

To identify the transported target parameters, we must meet the following additional criteria of conditional population exchangeability and population positivity [10]. We used a selection diagram to identify a set of measured characteristics that satisfy these assumptions. Selection diagrams are directed acyclic graphs that indicate both the causal model and where differences in the causal model might exist between the trial and the target populations [28, 29]. If there is a set of variables that, if conditioned on, will make the selection nodes independent from the outcome, then the conditional population exchangeability assumption is met and the trial effect can be transported [28, 30]. Figure 1 depicts our proposed selection diagram representing the assumed causal model within the ASSIST trial and assumed differences between the study population and each target population, with selection nodes indicating where these differences might exist and arrows indicating hypothesized causal relationships [28].

To transport the ASSIST trial results to the target populations we first appended each SDDU target population data set to the ASSIST trial data to create five separate appended and imputed data sets [31]. In the appended data sets all column variable names were the same, with ASSIST trial participants stacked in rows above the SDDU participants. Next, we estimated weights related to the inverse odds of selection into ASSIST, by fitting multi‐level logistic regression models (students nested within schools) for selection into ASSIST (0 = SDDU wave, 1 = ASSIST) in each of the five data sets based on participant gender identity, smoking status, ethnicity, age, age when they first smoked a cigarette, residence with a smoker and whether they had a lesson on cigarette smoking in the past 12 months, modelled as either binary or categorical variables.

To estimate the transported intention to treat effects of ASSIST, we applied the weights for selection into ASSIST for each SDDU year to multilevel logistic regression models (students nested within schools) with main terms for study arm adjusting for the school‐level stratification variables used in the ASSIST trial. Using this model, we estimated the odds ratios and accompanying 95% confidence intervals for weekly smoking in 2004, 2006, 2014, 2016 and 2021, and confidence interval ratios were used to compare precision across estimates. The confidence interval ratio is the ratio of the upper to the lower confidence limit [11].

To examine the impact of not imputing the data we conducted sensitivity analysis where we re‐ran the analysis in a complete case sample where participants with any missing data were excluded. As the trial was conducted in Wales and England but the target populations were only sampled from England, we also re‐ran the analysis after excluding schools from Wales.

Missing data were addressed through multiple imputation using chained equations. Missingness was assumed to be missing at random, conditional on the same set of self‐reported characteristics used to estimate differences between the trial and the target population. Missing data in the ASSIST trial and each target population data set are shown in Table S2. The plausibility of the missing‐at‐random assumption was tested by seeing whether the data we observed could predict a binary missing, or not, indicator variable on each of the variables used (0 = no missing data; 1 = data missing). Table S3 shows the results from a multi‐level logistic regression model suggesting gender, baseline smoking status and smoking status at the 2‐year follow‐up were associated with whether data were missing on students having received a lesson on smoking or not in the past year, suggesting the missing‐at‐random assumption is plausible. We used a multivariate normal imputation model to impute all missing data to create 20 imputed data sets. Data were imputed in the ASSIST trial data set and each SDDU data set separately. Results were obtained by pooling estimates across 20 imputed data sets using Rubin's rules [32, 33], with an assessment of Monte Carlo errors suggesting that this was a suitable number of imputations [34].

Analyses were not pre‐registered, and results should be considered as exploratory. All analyses were conducted using Stata 17.0 (StataCorp LLC, College Station, TX, USA) [35]. The code for these analyses can be found on GitHub [36].

## RESULTS

Figure S1 shows the trial profile. In the SDDU, 3958 students participated in 2004 (89% response), 3377 in 2006 (85% response), 3145 in 2014 (87%), 4874 in 2016 (93%) and 3587 in 2021 (92% response). Comparing the baseline characteristics of ASSIST trial participants and each target population, the trial had more: occasional, experimental and ex‐smokers (37.6% vs 25.2% in 2004 and 5.4% in 2021 in the SDDU); weekly smokers (4.1% vs 3.6% in 2003 and 0.2% in 2021 in the SDDU); students who lived with a smoker (51.7% vs 46.0% in 2004 and 28.1% in 2021 in the SDDU); 12‐year‐olds than 13‐year‐olds; and participants who smoked their first cigarette before 12 years of age. Moreover, the ASSIST trial had fewer participants from ethnic minorities (7.8% vs 14.4% in 2004 and 27.8%in 2021 in SDDU) (Tables 1 and S4).

**TABLE 1:** Characteristics of 8756 participants in the ASSIST trial in 2004, followed for 2 years, and of 18 941 young people in England, 2004–2021.

Characteristic	Trial participants % (no.)	English population % (no.)				
		2004	2006	2014	2016	2021
All	8756	3958	3377	3145	4874	3587
Gender						
Boy	50.5 (4420)	52.4 (2073)	50.8 (1661)	51.0 (1605)	49.1 (2393)	51.4 (1844)
Girl	49.5 (4336)	47.6 (1885)	49.2 (1716)	49.0 (1540)	50.9 (2481)	48.6 (1743)
Smoking status						
Never smoked	58.3 (5105)	71.2 (2818)	72.9 (2463)	80.7 (2538)	89.3 (4352)	94.4 (3386)
Occasional, experimental or ex‐smokers	37.6 (3294)	25.2 (998)	23.3 (788)	16.5 (519)	9.6 (468)	5.4 (194)
Weekly smoker^a^	4.1 (357)	3.6 (142)	3.0 (100)	2.8 (88)	1.1 (54)	0.2 (7)
Ethnicity						
White	92.2 (8073)	85.6 (3388)	86.9 (2935)	85.6 (2692)	87.3 (4255)	72.2 (2590)
Minority	7.8 (683)	14.4 (570)	13.1 (442)	14.4 (453)	12.7 (619)	27.8 (997)
Age						
12 years old	78.0 (6830)	49.9 (1976)	50.6 (1709)	50.6 (1591)	43.7 (2129)	44.7 (1605)
13 years old	22.0 (1926)	50.1 (1982)	49.4 (1668)	49.4 (1554)	56.3 (2745)	55.3 (1982)
Age first smoked a cigarette						
Never smoked	57.9 (5069)	68.2 (2699)	70.0 (2364)	77.3 (2431)	87.4 (4260)	92.9 (3332)
0–10 years old	15.8 (1383)	10.6 (420)	8.6 (290)	6.6 (208)	2.4 (117)	1.8 (65)
11 years old	13.9 (1217)	8.4 (332)	8.8 (297)	5.9 (186)	2.7 (132)	1.7 (61)
12 years old	11.6 (1016)	10.1 (400)	12.6 (426)	10.2 (320)	4.0 (194)	1.9 (68)
13 years old	0.08 (71)	2.7 (107)	0 (0)	0 (0)	3.5 (171)	1.7 (61)
Live with a smoker						
Yes	51.7 (4527)	46.0 (1821)	48.1 (1628)	40.8 (1283)	29.4 (1433)	28.1 (1008)
No	48.3 (4229)	54.0 (2137)	51.8 (1749)	59.2 (1862)	70.6 (3441)	71.9 (2579)
Lesson on smoking						
Yes	57.3 (5017)	58.7 (2323)	57.5 (1942)	60.3 (1896)	61.5 (2997)	62.8 (2253)
No	26.5 (2321)	31.7 (1255)	31.5 (1064)	28.6 (899)	22.3 (1087)	19.3 (692)
I do not know	16.2 (1418)	9.6 (380)	11.0 (371)	11.1 (350)	16.2 (790)	17.9 (642)

^a^ Weekly smoker defined as those who smoked at least one cigarette a week.

**FIGURE 2:**
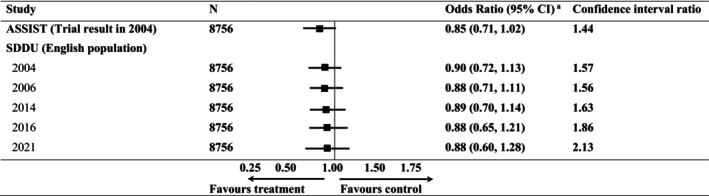
Odds ratios from a multi‐level model for intervention effect on smoking status in the ASSIST trial and young people in England, 2004–2021.

The odds ratio for the effect of ASSIST on weekly smoking at the 2‐year follow‐up reported in the original trial was 0.85 (95% CI = 0.71–1.02) [14]. After weighting the trial participants to the target population in 2004, the estimated odds ratio of the effect of ASSIST was 0.90 (95% CI = 0.72–1.13) (Figure 2). The estimated transported odds ratio remained stable over time. All transported odds ratios had wider confidence intervals than those in the trial, expressed in Figure 1 as confidence limit ratios. The wider interval widths reflect differences between the trial sample and target populations increasing over time. Results were similar when analyses were re‐run in the complete case sample (Table S5) and again when using only ASSIST trial schools from England (Table S6).

## DISCUSSION

These exploratory analyses suggest the effect of a school‐based smoking prevention intervention reported in the original trial may not have been replicated when rolled out in the target population. The transported odds ratios were slightly smaller than those found in the trial, and confidence intervals increased in width between 2004 and 2021, suggesting an increasing uncertainty in the effect of ASSIST over time.

### Comparisons with existing studies

To date, intervention effects have been transported to target populations [10, 11] to understand heterogeneity between trial sites [12] or population subgroups within a trial [13], but not in the same target population over time. These studies have found mixed evidence that intervention effects are transportable [10, 11, 12, 13]. The attenuation and loss in the precision of transported trial effects that we have found replicates the findings of studies estimating the effects of antiretroviral therapy to treat HIV/AIDS in the 2006 US population [11], and also rosuvastatin in the primary prevention of cardiovascular events in the UK general practice population [31]. Our analysis has extended these results by repeating analysis to explore changes in effects in the target population over the 17‐year period in which the intervention was licensed and delivered.

Our study is not the first to find that trial participants may not be representative of the population they are sampled from [1]. In 2004, when ASSIST ended, students in the trial were more likely to have smoked/currently smoke and to live with a smoker, and were less likely to be from an ethnic minority, than those in 2004 in the target population. Previous research has attributed differences between trial participants and target populations to the eligibility criteria used and variations in response rates across population subgroups. For example, in a review of protocols from 32 RCTs evaluating treatments for HIV [37], a median of 42% of women infected with HIV in a large observational cohort, the Women's Interagency HIV Study, would have been excluded. In contrast, in the ASSIST trial there were relatively few exclusion criteria and the student‐level response rates were 85–95% [14]. An alternative explanation is that in addition to changes in the context within which ASSIST was delivered, the ASSIST trial sampled a smaller number of schools (the ASSIST sample frame included 223 schools; the SDDU sample frame had 450 schools in 2004 and 1023 schools in 2021) and from a smaller geographic area that the SDDU, which also reduced the representativeness of the trial sample compared with the target population.

Another contextual difference between the trial and the target population is the tobacco control measures in place in the UK. Since the ASSIST trial ended in 2002, the UK has seen a ban on smoking in public places [38], the EU Tobacco Products Directive placed limits on the maximum cigarette pack size and mandated health warnings on tobacco products, there has been a ban on tobacco vending machines [39], increases in the legal age to purchase tobacco from 16 to 18 years [40] and reductions in the affordability of tobacco [41], along with advent of e‐cigarettes/vaporized nicotine products. That the confidence interval ratio increased over time reflects an increasing ‘distance’ between the trial participants and the target population that coincided with the strengthening in tobacco control measures in the UK [38, 39, 40, 41].

### Strengths and weaknesses

The strengths of this study are that it is the first to estimate the effect of a school‐based intervention in its intended target population over time. Our re‐weighting of the trial population allowed us to estimate the likely treatment effect in the target population over the 17‐year period during which ASSIST was being rolled out, while still benefitting from the advantages of randomization. The application of transportability relies on the availability of individual‐level data in both the trial and the target populations. For transportability assumptions to be reasonably met these data need to include relevant effect modifiers in the trial and the target population. These are not trivial requirements, but as population‐level surveys are routinely conducted in many countries [42, 43], and surveys and trials start to use harmonized core outcome sets, transportability could become more widely used to inform decisions on whether to commission or decommission interventions in addiction research.

There are, however, a number of limitations that need to be considered when interpreting our results. Akin to the assumption of no unmeasured confounding in observational studies, the estimated transported odds ratios assume we adequately accounted for and modelled the joint distribution of characteristics that might modify the effectiveness of ASSIST and predict selection into the trial. In any estimation of transportability estimates there are likely to be omitted effect modifiers, and our analysis is no different. For example, there was no harmonized measure of socio‐economic status available in the trial and SDDU data sets we used. The imputation of missing values would have increased the volume of data available to predict selection and effect modification. That said, in practice, at best only a subset of the characteristics that lead to effect heterogeneity will be available, and a realistic estimate of transportation will need to be qualified by additional work on factors that might have affected mechanisms underpinning the effectiveness of ASSIST in England over time. In both the ASSIST trial and the SDDU data set there was evidence of measurement error. There was a difference between the number of self‐reported never smokers in the questions on smoking status and the age at which participants had first smoked. This measurement error is likely to reflect a combination of recall bias, changes in social desirability and the interpretation of questions [44]. These analyses were not pre‐registered and require independent confirmatory studies, ideally with data sets that can better characterize the distribution of effect modifiers between the trial and the target populations.

## CONCLUSION

Our findings suggest the effects of a school‐based smoking prevention intervention reported in the original trial may not have been replicated during the 17‐year period of commissioning and delivery in the target population. Transportability is a transparent, efficient and underused framework that could be used to help inform decisions on whether to commission a new intervention, particularly when contrasted with the alternative of repeating the trial. Researchers publishing trial results should ensure data sets are made available so commissioners can generate tailored estimates of effectiveness within their population before deciding whether to fund a new intervention.

## AUTHOR CONTRIBUTIONS

**James White:** Conceptualization; formal analysis; writing—original draft.

## ACKNOWLEGDEMENTS

J.W. would like to thank Prof. Rhian Daniel for her comments on a draft of this article.

## DECLARATION OF INTERESTS

J.W. is a scientific adviser to Evidence to Impact (http://evidencetoimpact.com), a not‐for‐profit organization that licenses ASSIST.

## CLINICAL TRIAL REGISTRATION

Current Controlled Trials ISRCTN 55572965.

## Supporting information


**Figure S1.** CONSORT flow diagram.
**Table S1.** Stages in the ASSIST intervention.
**Table S2.** Characteristics of the 8756 participants in the ASSIST trial in 2004 followed for 2 years and of the 18 941 young people in England, 2004–2021.
**Table S3.** Association between observed data and whether data are missing or not on the having had a lesson on smoking in the past year variable.
**Table S4.** Odds ratios (95% confidence intervals) for selection into the ASSIST trial in 2004 for young people in England in 2004–2021.
**Table S5.** Odds ratios and risk differences with 95% confidence intervals for weekly smoking at a 2‐year follow‐up for participants in the ASSIST trial in 2004 and for young people in England 2004–2021 in the complete case sample.
**Table S6.** Odds ratios and risk differences with 95% confidence intervals for weekly smoking at a 2‐year follow‐up for participants in the ASSIST trial in 2004 and for young people in England 2004–2021 in students.

## Data Availability

Data from the Smoking, Drinking and Drug Use Among Young People in England Surveys are available to researchers upon application (https://discover.ukdataservice.ac.uk). Applicants interested in requesting trial data should apply via the data application form available on the Centre for Trials Research website (https://www.cardiff.ac.uk/centre-for-trials-research/collaborate-with-us/data-requests). In the first instance, enquiries about access to the trial data should be addressed to Prof. James White, School of Medicine, Cardiff University.
