# Supplementary material for: Transporting the effect of the ASSIST school‐based smoking prevention intervention to the Smoking, Drinking and Drug Use Among Young People in England Survey (2004–2021): A secondary analysis of a randomized controlled trial
Source: Addiction. 2025 Jul 7;120(11):2223–30. doi: 10.1111/add.70141 (PMC12529232; doi:10.1111/add.70141)
Supplement: Supplementary file 1 — Figure S1. CONSORT flow diagram. Table S1. Stages in the ASSIST intervention. Table S2. Characteristics of the 8756 participants in the ASSIST trial in 2004 followed for 2 years and of the 18 941 young people in England, 2004–2021. Table S3. Association between observed data and whether data are missing or not on the having had a lesson on smoking in the past year variable. Table S4. Odds ratios (95% confidence intervals) for selection into the ASSIST trial in 2004 for young people in England in 2004–2021. Table S5. Odds ratios and risk differences with 95% confidence intervals for weekly smoking at a 2‐year follow‐up for participants in the ASSIST trial in 2004 and for young people in England 2004–2021 in the complete case sample. Table S6. Odds ratios and risk differences with 95% confidence intervals for weekly smoking at a 2‐year follow‐up for participants in the ASSIST trial in 2004 and for young people in England 2004–2021 in students. [file ADD-120-2223-s001.docx]

**Supplementary information**

1. Figure S1. CONSORT flow diagram
2. Table S1. Stages in the ASSIST intervention
3. Table S2. Characteristics of 8,756 participants in the ASSIST trial in 2004 followed for 2-years and of 18,941 young people in England, 2004-2021
4. Table S3. Association between observed data and whether data are missing or not on the having had a lesson on smoking in the past year variable
5. Table S4. Odds ratios (95% confidence intervals) for selection into the ASSIST trial in 2004 for young people in England in 2004-2021
6. Table S5. Odds ratios and risk differences with 95% confidence intervals for weekly smoking at a 2-year follow-up for participants in the ASSIST trial in 2004 and for young people in England 2004-2021 in the complete case sample
7. Table S6. Odds ratios and risk differences with 95% confidence intervals for weekly smoking at a 2-year follow-up for participants in the ASSIST trial in 2004 and for young people in England 2004-2021 in students

127 visits to schools by research team

223 potentially eligible schools invited

5372 eligible students

5074 (95%) participated

5358 eligible students

5187 (97%) participated

5562 eligible students in 29 control schools

5481 eligible students in 30 intervention schools

5087 (95%) analysed

190 withdrawn by parents

123 withdrawn by parents

7 schools withdrew

6 schools withdrew;

8 ineligible;

47 schools not selected at random

96 schools not interested

59 committed at agreement stage

66 randomized

**Baseline data collection**

58 students left study

4 students join study

55 students left study

10 students join study

4950 (92%) analysed

5303 eligible students

4774 (90%) analysed

**After intervention follow-up**

5308 eligible students

4509 (85%) analysed

232 students left study

29 moved to intervention school

212 students join study

47 moved from intervention school

**1-year follow-up**

5296 eligible students

4629 (87%) analysed

5306 eligible students

4333 (82%) analysed

147 students left study

47 moved to a control school

158 students join study

29 moved from a control school

158 students left study

6 moved to a control school

140 students join study

11 moved from a control school

239 students left study

11 moved to an intervention school

212 students join study

6 moved from an intervention school

**2-year follow-up**

5283 eligible students

4563 (86%) in the transportability analysis

5274 eligible students

4193 (80%) in the transportability analysis

**Figure S1. CONSORT flow diagram**

**Table S1. Stages in the ASSIST intervention**

| **Nomination of peer supporters**   - Students aged 12–13 years (UK Year 8) were asked to identify influential peers using three questions, “Who do you respect in Year 8 at your school?”, “Who are good leaders in sports or other groups activities in Year 8 at your school?”, and “Who do you look up to in Year 8 at your school?” - The 18% of year 8 pupils receiving the most peer nominations were invited to a recruitment meeting. |
| --- |
| **Recruitment of peer supporters**   - A meeting was held with nominees to explain the role of a peer supporter and answer questions. - Trainers made it clear that students who smoked could only be peer supporters if they commit to trying to stop smoking. |
| **Training of peer supporters**   - Training was held off the school site over two days and delivered by a team of external trainers experienced in youth work and health-promotion. - The aims of the training were to: provide information about risks of smoking and benefits of remaining smoke-free; develop communication skills including, listening, cooperation and negotiation, and conflict resolution; enhance students’ confidence, empathy, assertiveness, attitudes to risk-taking, and exploration of personal values. |
| **Intervention period**   - Ten-week peer-led intervention during which peer supporters had informal conversations with their peers about smoking (for example, when travelling to and from school, in breaks, at lunchtime, and after school in their free-time), and logged conversations in a pro-forma diary. - Four school-based follow-up visits with peer supporters and led by ASSIST trainers aimed to provide support and guidance. |
| **Acknowledgment of peer supporters’ contribution**   - All peer supporters were presented with a certificate. - Peer supporters who handed in their diary were presented with a gift certificate. |

**Table S2. Characteristics of 8,756 participants in the ASSIST trial in 2004 followed for 2-years and of 18,941 young people in England, 2004-2021**

| **Characteristic** | **Trial participants, % (No.)** |  | **English population, % (No.)** | | | | |
| --- | --- | --- | --- | --- | --- | --- | --- |
|  |  |  | **2004** | **2006** | **2014** | **2016** | **2021** |
| All | 8,756 |  | 3,958 | 3,377 | 3,145 | 4,874 | 3,587 |
| Gender |  |  |  |  |  |  |  |
| Boy | 50.5 (4,420) |  | 52.4 (2,073) | 50.8 (1,661) | 51.0 (1,605) | 48.9 (2,383) | 50.7 (1,819) |
| Girl | 49.5 (4,336) |  | 47.6 (1,885) | 49.2 (1,716) | 49.0 (1,540) | 50.5 (1,540) | 48.0 (1,720) |
| Missing | 0 (0) | (0) | 0 (0) | 0 (0) | 0 (0) | 0.6 (31) | 1.3 (48) |
| Smoking status |  |  |  |  |  |  |  |
| Never smoked | 58.3 (5,105) |  | 71.1 (2,815) | 72.9 (2,463) | 80.1 (2,519) | 88.0 (4,290) | 92.6 (3,322) |
| Occasional, experimental,  or ex-smokers | 37.6 (3,294) |  | 24.3 (960) | 23.3 (788) | 16.5 (518) | 9.6 (470) | 5.4 (193) |
| Weekly smoker ^a^ | 4.1 (357) |  | 3.6 (142) | 3.0 (100) | 2.8 (88) | 1.1 (52) | 0.2 (6) |
| Missing | 0 (0) |  | 1.0 (41) | 0.8 (26) | 0.6 (20) | 1.3 (62) | 1.8 (66) |
| Ethnicity |  |  |  |  |  |  |  |
| White | 91.0 (7,971) |  | 85.0 (3,958) | 81.7 (2,760) | 80.9 (2,543) | 79.5 (3,874) | 65.9 (2,365) |
| Mixed race | 3.3 (288) |  | 3.7 (147) | 3.3 (112) | 3.8 (120) | 3.0 (145) | 3.7 (131) |
| Asian or Asian British | 2.0 (173) |  | 6.0 (236) | 5.4 (181) | 5.2 (163) | 5.6 (274) | 13.8 (496) |
| Black or Black British | 1.1 (97) |  | 2.7 (108) | 3.1 (106) | 3.8 (121) | 2.6 (125) | 3.8 (135) |
| Other | 1.3 (113) |  | 1.9 (76) | 0.6 (20) | 0.9 (27) | 0.4 (20) | 4.1 (145) |
| Missing | 1.3 (114) |  | 0.7 (27) | 5.9 (198) | 5.4 (171) | 8.9 (436) | 8.8 (315) |
| Age |  |  |  |  |  |  |  |
| 10 years old | 0 (0) |  | 0 (0) | 0 (0) | 0 (0) | 0 (0) | 0 (0) |
| 11 years old | 0.1 (11) |  | 0 (0) | 0 (0) | 0 (0) | 0 (0) | 0 (0) |
| 12 years old | 77.3 (6,768) |  | 49.9 (1,976) | 50.6 (1,709) | 50.6 (1,591) | 43.7 (2,128) | 44.7 (1,605) |
| 13 years old | 22.0 (1,922) |  | 50.1 (1,982) | 49.4 (1,668) | 49.4 (1,554) | 56.3 (2,746) | 55.3 (1,982) |
| 14 years old | 0.1 (8) |  | 0 (0) | 0 (0) | 0 (0) | 0 (0) | 0 (0) |
| Missing | 0.5 (47) |  | 0 (0) | 0 (0) | 0 (0) | 0 (0) | 0 (0) |
| Age 1^st^ smoked a cigarette |  |  |  |  |  |  |  |
| Never smoked | 57.9 (5,069) |  | 68.1 (2,695) | 70.0 (2,363) | 77.3 (2,432) | 86.7 (4,224) | 91.7 (2,363) |
| 0-10 years old | 15.8 (1,383) |  | 10.0 (395) | 8.1 (274) | 6.1 (193) | 2.0 (97) | 1.3 (47) |
| 11 years old | 13.9 (1,218) |  | 8.0 (316) | 8.3 (280) | 5.5 (174) | 2.3 (113) | 1.4 (49) |
| 12 years old | 11.6 (1,018) |  | 9.6 (380) | 8.7 (293) | 7.0 (219) | 3.8 (185) | 1.7 (62) |
| 13 years old | 0.7 (59) |  | 2.6 (103) | 3.1 (106) | 2.8 (87) | 3.4 (167) | 1.6 (59) |
| 14 years old | 0 (0) |  | 0 (0) | 0.0 (0) | 0 (0) | 0.0 (0) | 0.0 (0) |
| Missing | 0.1 (9) |  | 1.7 (69) | 1.7 (61) | 1.3 (40) | 1.8 (88) | 2.3 (82) |
| Live with a smoker |  |  |  |  |  |  |  |
| Yes | 51.1 (4,470) |  | 44.5 (1,763) | 46.2 (1,560) | 38.9 (1,222) | 29.1 (726) | 23.9 (451) |
| No | 47.7 (4,183) |  | 52.3 (2,069) | 49.7 (1,679) | 56.7 (1,784) | 70.9 (1,768) | 62.6 (1,181) |
| Missing | 1.2 (108) |  | 3.2 (126) | 4.1 (138) | 4.4 (139) | 0 (0) | 13.5 (255) |
| Not applicable ^b^ | - |  | - | - | - | 2,380 | 1,700 |
| Lesson on smoking |  |  |  |  |  |  |  |
| Yes | 55.0 (4,814) |  | 57.1 (2,260) | 55.1 (1,859) | 58.4 (1,838) | 57.6 (2,805) | 56.5 (2,028) |
| No | 25.4 (2,225) |  | 30.7 (1,215) | 30.1 (1,017) | 27.7 (870) | 20.8 (1,015) | 17.4 (623) |
| I don’t know | 15.6 (1,364) |  | 9.3 (370) | 10.5 (356) | 10.7 (336) | 15.1 (738) | 16.0 (572) |
| Missing | 4.0 (1,364) |  | 2.9 (113) | 4.3 (145) | 3.2 (101) | 6.5 (316) | 10.2 (364) |

^a^ Weekly smoker defined as those who smoked at least ≥1 cigarette a week. ^b^ In the 2016 and 2021 Smoking, Drinking and Drug Use Survey some students were routed to questions so some were unable this question.

**Table S3. Association between observed data and whether data are missing or not on the having had a lesson on smoking in the past year variable**

| **Variable** | **Odds Ratio (95% CI)** |
| --- | --- |
| Gender |  |
| Boy |  |
| Girl | 0.60 (0.48, 0.75) |
| Baseline smoking status |  |
| Never smoked |  |
| Weekly smoker ^a^ | 1.99 (1.25, 3.17) |
| 2-year follow-up smoking status |  |
| Never smoked |  |
| Weekly smoker ^a^ | 1.66 (1.26, 2.19) |

^a^ Smoking at least one cigarette a week; Reference categories: boy, never smoker

**Table S4. Odds ratios (95% confidence intervals) for selection into the ASSIST trial in 2004 for young people in England in 2004-2021**

| **Characteristic** | **English population ^a^** | | | | |
| --- | --- | --- | --- | --- | --- |
|  | **2004 (n = 3,985)** | **2006 (n = 3,377)** | **2014 (n = 3,145)** | **2016 (n = 4,874)** | **2021 (n = 3,587)** |
| Gender |  |  |  |  |  |
| Boy | Reference category | - | - | - | - |
| Girl | 1.00 (0.92, 1.08) | 0.90 (0.82, 0.98) | 0.95 (0.87, 1.04) | 0.92 (0.85, 1.00) | 1.00 (0.91, 1.10) |
| Smoking status |  |  |  |  |  |
| Never smoked | Reference category | - | - | - | - |
| Occasional, experimental,  or ex-smokers | 3.62 (2.82, 4.64) | 4.40 (3.41, 5.68) | 5.53 (4.22, 7.25) | 7.06 (5.20, 9.59) | 8.34 (5.66, 12.27) |
| Weekly smoker | 2.97 (2.16, 4.06) | 4.10 (2.92, 5.75) | 3.67 (2.57, 5.25) | 5.79 (3.77, 8.90) | 26.53 (10.78, 65.28) |
| Ethnicity |  |  |  |  |  |
| White | Reference | - | - | - | - |
| Ethnic minority | 0.51 (0.45, 0.58) | 0.57 (0.49, 0.65) | 0.52 (0.45, 0.59) | 0.59 (0.51, 0.67) | 0.23 (0.20, 0.26) |
| Age |  |  |  |  |  |
| 12 years old | Reference | - | - | - | - |
| 13 years old | 0.28 (0.26, 0.30) | 0.29 (0.27, 0.32) | 0.29 (0.26, 0.32) | 0.22 (0.21, 0.24) | 0.23 (0.21, 0.25) |
| Age 1^st^ smoked a cigarette |  |  |  |  |  |
| Never smoked | Reference | - | - | - | - |
| 0-10 years old | 0.56 (0.43, 0.72) | 0.58 (0.44, 0.77) | 0.71 (0.53, 0.95) | 1.55 (1.12, 2.15) | 1.77 (1.15, 2.72) |
| 11 years old | 0.55 (0.42, 0.72) | 0.46 (0.35, 0.61) | 0.62 (0.47, 0.84) | 1.06 (0.76, 1.48) | 1.47 (0.95, 2.26) |
| 12 years old | 0.49 (0.37, 0.64) | 0.47 (0.35, 0.61) | 0.52 (0.39, 0.70) | 0.74 (0.53, 1.04) | 1.41 (0.91, 2.18) |
| 13 years old | 0.20 (0.13, 0.30) | 0.13 (0.09, 0.20) | 0.15 (0.09, 0.23) | 0.09 (0.06, 0.15) | 0.17 (0.10, 0.29) |
| Live with a smoker |  |  |  |  |  |
| No | Reference | - | - | - | - |
| Yes | 1.11 (1.02, 1.20) | 1.00 (0.91, 1.09) | 1.23 (1.12, 1.35) | 1.85 (1.66, 2.07) | 1.78 (1.56, 2.04) |
| Lesson on smoking |  |  |  |  |  |
| Yes | Reference | - | - | - | - |
| No | 0.79 (0.72, 0.87) | 0.78 (0.71, 0.86) | 0.90 (0.82, 1.00) | 1.10 (0.99, 1.22) | 1.40 (1.25, 1.58) |
| I don’t know | 1.67 (1.46, 1.90) | 1.43 (1.25, 1.64) | 1.46 (1.27, 1.67) | 0.97 (0.86, 1.09) | 0.92 (0.81, 1.05) |

^a^ Sample size deriving estimates comprised the ASSIST 2-year estimate trial population (n = 8756) plus the respective SDDU year participants (e.g., SDDU 2004 = 12,741).

**Table S5. Odds ratios and risk differences with 95% confidence intervals for weekly smoking at a 2-year follow-up for participants in the ASSIST trial in 2004 and for young people in England 2004-2021 in the complete case sample**

| **Study** | **N** | **Odds ratio (95% CI)** | **Confidence interval ratio** |
| --- | --- | --- | --- |
| ASSIST (Trial result in 2004) | 8756 | 0.85 (0.71, 1.02) | 1.44 |
| SDDU (Population results) |  |  |  |
| 2004 | 8184 | 0.89 (0.71, 1.11) | 1.56 |
| 2006 | 8184 | 0.89 (0.71, 1.12) | 1.58 |
| 2014 | 8184 | 0.88 (0.73, 1.07) | 1.47 |
| 2016 | 8184 | 0.98 (0.72, 1.35) | 1.88 |
| 2021 | 8184 | 0.84 (0.57, 1.25) | 2.19 |

SDDU: Smoking, Drinking and Drug Use Study

**Table S6. Odds ratios and risk differences with 95% confidence intervals for weekly smoking at a 2-year follow-up for participants in the ASSIST trial in 2004 and for young people in England 2004-2021 in students in schools in England**

| **Study** | **N** | **Odds ratio (95% CI)** | **Confidence interval ratio** |
| --- | --- | --- | --- |
| ASSIST (Trial result in 2004) | 8756 | 0.94 (0.76, 1.16) | 1.53 |
| SDDU (Population results) |  |  |  |
| 2004 | 4702 | 0.96 (0.72, 1.28) | 1.78 |
| 2006 | 4702 | 0.95 (0.71, 1.26) | 1.77 |
| 2014 | 4702 | 0.96 (0.70, 1.31) | 1.87 |
| 2016 | 4702 | 0.90 (0.61, 1.31) | 2.15 |
| 2021 | 4702 | 0.90 (0.54, 1.48) | 2.74 |

SDDU: Smoking, Drinking and Drug Use Study
